# Genetic instability in lung cancer: concurrent analysis of chromosomal, mini- and microsatellite instability and loss of heterozygosity

**DOI:** 10.1038/sj.bjc.6603121

**Published:** 2006-04-25

**Authors:** H Ninomiya, K Nomura, Y Satoh, S Okumura, K Nakagawa, M Fujiwara, E Tsuchiya, Y Ishikawa

**Affiliations:** 1 Department of Pathology, The Cancer Institute, Japanese Foundation for Cancer Research (JFCR), 3-10-6 Ariake, Koto-ku, Tokyo 135-8550, Japan; 2 Department of Chest Surgery, The Cancer Institute Hospital, Japanese Foundation for Cancer Research (JFCR), Tokyo, Japan; 3 Department of Pathology, Japanese Red Cross Medical Center, Tokyo, Japan; 4 Kanagawa Cancer Center, Kanagawa, Japan

**Keywords:** minisatellite instability, microsatellite instability, loss of heterozygosity (LOH), chromosome instability, lung cancer

## Abstract

To investigate what kind of genetic instability plays important roles in lung carcinogenesis, we analyzed micro- and minisatellite instability, loss of heterozygosity (LOH) and chromosome instability in 55 cases of lung cancer, including, 10 squamous cell, 5 large cell, and 3 small cell carcinomas, and 37 adenocarcinomas. Analysis of minisatellite instability, the mechanism of which is different from microsatellite instability, has not been reported previously. Minisatellite instability was detected in only one case (1/55, 1.8%), and the frequency of microsatellite instability was low, being found only in three cases (3/55, 5.5%). In contrast, LOH, for at least in one locus, was observed in 27 cases (49.1%). In adenocarcinomas, the frequency of LOH was higher in poorly differentiated compared to more differentiated carcinomas. For chromosome instability, a similar correlation between differentiation grade and instability was observed in adenocarcinomas. And instability was more common in large cell and small cell carcinomas than in adenocarcinomas. Our analysis showed that chromosome instability and LOH, rather than mini- and microsatellite instability, play significant roles in the development of lung cancer.

Despite advances in diagnosis and treatment, lung cancer remains a major cause of cancer mortality worldwide. Accumulation of multiple genetic alterations is known to be associated with the development and progression of cancer (Loeb *et al*, 1991). The accumulation of these alterations is often driven by mutator phenotypes. For example, in hereditary non-polyposis colorectal cancer (HNPCC), it has been demonstrated that a mismatch repair gene deficit leads to microsatellite instability that plays a major role in the development of cancer (Aaltonen *et al*, 1993). It has been suggested that microsatellite instability may cause nucleotide-level mutations such as point mutations that may facilitate the inactivation of tumour suppressor genes, such as p53, resulting in dysregulation of the cell cycle and apoptosis (Hollstein *et al*, 1991). In addition to these nucleotide-level mutations, chromosome instability, which includes deletion, translocations, duplications and inversions, provides another level of disruption. When chromosomal instability occurs in genomic regions coding tumour suppressor or DNA repair genes, it may be associated with the pathogenesis of cancer (Mitelman *et al*, 1997). Loss of heterozygosity (LOH) analysis is often performed to examine deletion, duplication and recombination. Loss of heterozygosity (LOH) is frequently seen in cancer cells and is thought to occur through genetic instability at a chromosomal or similar level. As distinct pathways in the nucleotide level and the chromosomal level mutations have been suggested in different cancers (Lengauer *et al*, 1998), it is important to determine which pathway plays more important roles in cancers of interest.

Minisatellite instability has been observed in descendants of individuals exposed to low-dosage radiation following the Chernobyl nuclear plant accident (Dubrova, 1996). Micro- and minisatellites are similar in repetitive structure, but suggested to differ in the mechanisms for destabilization (Lopes, 2002). To our knowledge, there has been no report on minisatellite instability in lung cancers.

We previously investigated the difference between allelotypes of squamous cell carcinoma (SCC) and adenocarcinoma (AC) and the relationship between frequency of LOH and differentiation grades of AC (Tsuchiya *et al*, 1992; Sato *et al*, 1994; Ishikawa *et al*, 2002). In the current study, we examined the frequency of minisatellite instability and compared it with those of microsatellite instability, chromosome instability and LOH in four major histological types of lung cancer.

## MATERIALS AND METHODS

### Cases and histological diagnosis

Lung cancers and corresponding normal tissues were obtained from 55 individuals, surgically operated for lung cancer from 1996 to 1997 at the Cancer Institute Hospital of the Japanese Foundation for Cancer Research, Tokyo. Histological diagnosis was made using sections through the largest cut surface of each tumour stained by hematoxylin-eosin, alcian blue, periodic acid Schiff reaction and Elastica van Gieson for elastic fibers. Based on the World Health Organization classification (Travis *et al*, 1999) histology of tumours were as follows: 37 AC, composed of 19 well differentiated (w/d), 10 moderately differentiated (m/d) and 8 poorly differentiated (p/d) carcinomas as well as 10 SCC, 5 large cell carcinomas (LCC), and 3 small cell carcinomas (SCLC). Clinical characteristics are shown in Table 1.

Fresh tissue samples, taken from primary tumours and corresponding normal lung, were snap-frozen in liquid nitrogen within 20 min of resection and stored at –80°C before DNA extraction. Formalin-fixed paraffin-embedded sections were prepared for pathological examination. DNA was extracted from the bulk tissues of each specimen. At the time the tissues were snap-frozen, tumour specimens were cut into two, one was frozen for storage and the other was confirmed microscopically to contain significant amounts of cancer cells.

### Analysis of mini- and microsatellite instability

**Figure 1:**
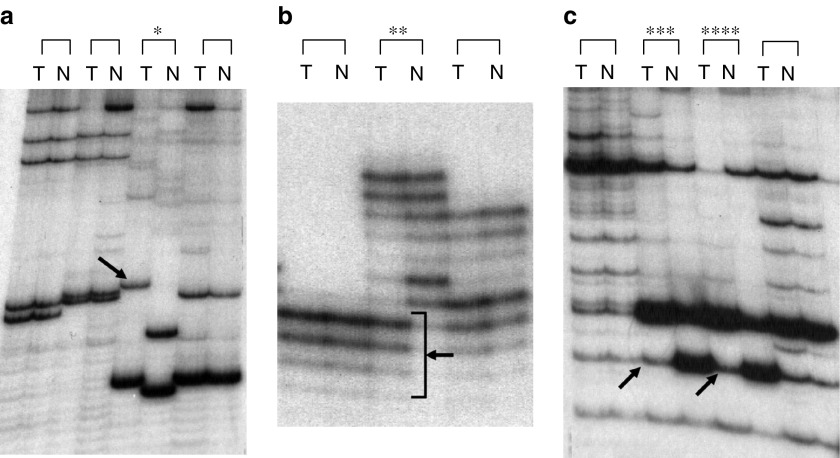
Representation of mini- and microsatellite instability and LOH. (**A**) Minisatellite instability at marker D1S80 (^*^Case 15 of well differentiated adenocarcinoma.). (**B**) Microsatellite instability at marker TP53 (^**^Case 2 of SCLC.). (**C**) LOH at marker D17S30 (^***^Case 6, ^****^Case 7 of poorly differentiated adenocarcinoma.) *Arrows*: alteration allele; T: tumour; N: normal tissue.

Three different loci, D1S80 (1p35–36), ApoB (2p24), D17S30 (17p13) were used for minisatellite analysis and six different loci, D1S226 (AFM184Xe; 1p21), D3S1067(3p21.1-3p23), D13S270 (13q14), TP53 (17p13.1), HPOCA3 (17p13.1) and D17S786 (17p13) for microsatellite analysis. Tumour and normal tissue DNA was amplified by polymerase chain reaction (PCR). A volume of 1.0 *μ*l of genomic DNA templates was suspended in a total volume of 20.0 *μ*l PCR mixture containing 12.0 *μ*l distilled H_2_O, 10 × PCR buffer, 2.0 *μ*l dNTP (2 mM), 0.10 *μ*l of ^32^P-labelled dCTP, 15 pmol of each primers and 0.90 *μ*l Taq DNA polymerase (Promega, Madison, WI, USA) and Taq start antibody with diluted buffer. Polymerase chain reaction amplification was performed with a hot start at 94°C for 5 min followed by 35 cycles of amplification at 94°C for 40 s, annealing at 60°C for 1 min, and extension at 72°C for 7 min. A final extension at 72°C was added for 7 min. Amplified PCR products were separated on a 6% polyacrylamide gel with a labelled marker for minisatellites, and on a urea gel for microsatellites. Electrophoresis was conducted at 400 V overnight for the former, and at 1500 V for 1 h for the latter. Then gels were exposed to X-ray films. The results were interpreted visually by two independent observers (YI and KN). Discrepancies were resolved by discussion. Tumour mini- and microsatellite instability appeared as additional bands and differed in size compared to normal tissue DNA. Allelic imbalance (i.e. LOH) was determined by the decrease in intensity of one allele relative to the other as compared between tumour and normal bands by visual inspection (Figure 1).

### Chromosome instability

Karyotyping by a conventional chromosome aberration analysis using Quinaqcrine-banding was performed after, typically, a few-week-long primary culture. Analysis of 10 cultured cells was intended. In cases where we could not obtain 10 metaphase cells, evaluated cells were fewer. Degrees of abnormality were subclassified to four groups by the number of structural alterations: 0, normal karyotype; L, less than 5; M, 6–15; H, more than 16. Only when the growth of fibroblastic cells and lymphocytes was apparent, we did not perform the analysis and denoted the sample as ‘n.p. (not proliferative)’. When slightly spindle cells grew and they could be tumour cells, or when samples showed ‘two cell patterns’ of apparently epithelial cells and slightly spindle and possibly tumour cells, we did analyse. Therefore, cells grown in culture denoted as 0 may contain fibroblasts or lymphocytes.

## RESULTS

### Minisatellite instability

**Figure 2:**
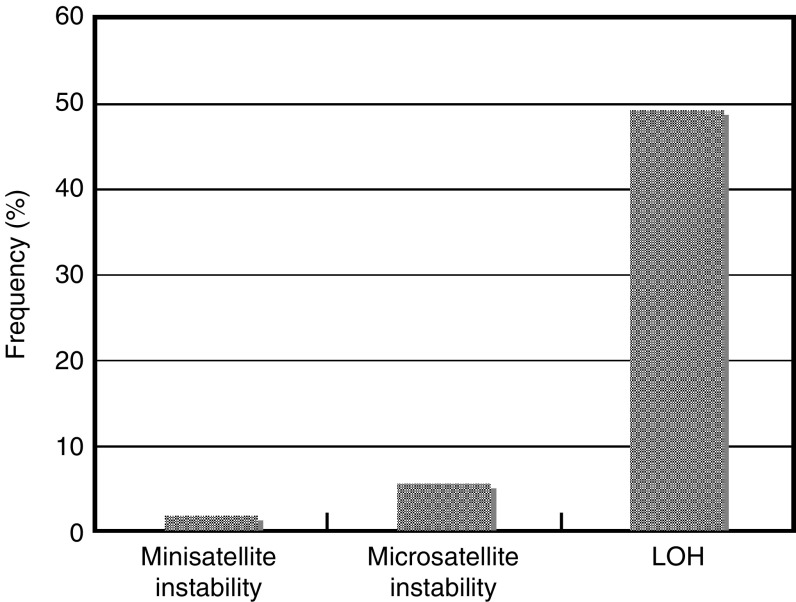
Comparison of frequency of genetic instability.

Of 55 cases of lung cancer, only one case (1.8%) (Figure 2, Case 15 of Table 2) of AC showed minisatellite instability in two of three investigated loci, D1S80 and ApoB (Table 3). This case also exhibited microsatellite instability in the four loci of markers (AFM184Xe, D3S1067, TP53 and D17S786) (Table 3).

### Microsatellite instability

Three cases (5.5%), including two ACs and one SCLC, exhibited microsatellite instability (Table 3). One AC showed the instability in loci of AFM184Xe, D3S1067, TP53 and D17S786, and the other AC in one locus, D3S1067. The SCLC case exhibited in the TP53 locus.

### LOH and fractional allelic loss

The incidence of LOH for at least in one locus was 27/55 (49.1%) (Figure 2). Detailed frequencies in pathological subtypes were 5/19 (26.3%) in w/d AC, 6/10 (60%) in m/d AC, 5/8 (62.5%) in p/d AC, 4/10 (40%) in SCC, 4/5 (80%) in LCC, and 3/3 (100%) in SCLC (Table 4). Loss of heterozygosity (LOH) was observed in loci of eight of nine markers except for D1S80. Higher frequency of LOH was found for TP53 (16 of 54 informative cases, 30.2%), HPOCA3 (21.8%), D17S786 (21.3%) than the others (Table 5). Fractional allelic loss (FAL), defined by a ratio of (numbers of alleles showing LOH)/(numbers of informative alleles) for each case, also significantly increased in SCLC (Table 4).

### Chromosome instability

**Figure 3:**
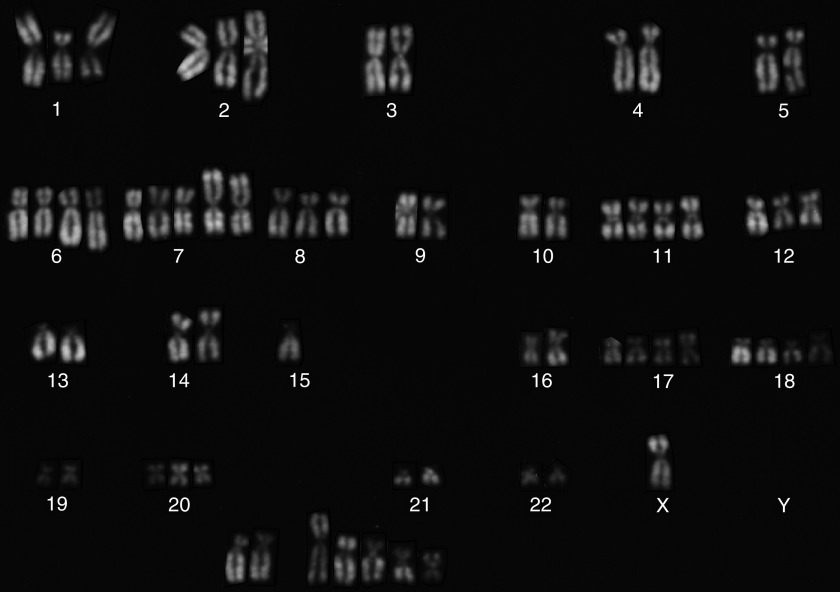
Typical karyotype of squamous cell carcinoma (Case 6); 67∼71<2n>,X,−Y,del(1)(p22),+del(1)(q32),+2,+2,+add(6)(q21) × 2,+7,+add(7)(p15) × 2,+8,+11,+11,+del(12)(q22) × 2,+13,add(14)(p13) × 2,−15,+17,+i(17)(q10),+18,+18,+20,+mar1 × 2,+4∼10mar[cp6].

Chromosome alteration was subclassified into four categories as mentioned above. Karyotype of each case was so unique and complex that we could not describe coherent tendency of structural rearrangement. Typical karyotype of SCC was demonstrated in Figure 3. In other histological types, karyotype of one typical case was shown below:

AC, w/d: Case 9:
 61∼65,XX,der(14;22)(q10;q10) × 2,+mar1 × 2,inc[cp2]/46,XX[2]
AC, m/d: Case 5:
 64∼74,inc[cp3]
AC, p/d: Case 1:
 47∼48,XY,del(1),(p22p32),+2,+8,−10,del(12)(q22),−14,der(15)t(1;15)(q12;p11),add(18)(q21),+1∼2r[cp10]
LCC: Case 5:
 52∼57,XY,t(1;10)(p11;p11),del(3)(p12),del(5)(p13),+6,del(6)(q15),add(7)(p11),add(8)(q24),del(8)(p12),add(9)(p13),add(11)(q23),add(12)(p13),der(13;14)(q10;q10),−14,add(14)(p13),−16,−19,−21,+mar1,+mar2,+mar3,+mar4,+6∼13mar[cp10]
SCLC: Case 2:
 76∼82<4n>,XXYY,del(1)(p31),−2,add(2)(q37) × 2,−3,−3,−3,i(3)(q10),4,−4,−4,−4,−5,−5,−5,−5,−7,−7,−10,−10,add(11)(p15),add(11)(q21) × 2,add(12)(q24) × 2,−13,−13,−16,−16,−19,−20,−21,−22,−22,+mar1,+mar2,+mar3 × 2,+mar4 × 2,+mar5 × 2,+mar6,+2∼7mar[cp10]

More than half of the cases of p/d AC (83.4% of cases with cell proliferation), SCC (55.5%), LCC (75.0%) and SCLC (100%) exhibited moderate to high degrees of instability (Table 6).

## DISCUSSION

Correlations between genetic instability and tumour development have been investigated extensively. In this study, the frequency of mini- and microsatellite instability, LOH and chromosome instability were elucidated for major histological types of lung cancer. To our knowledge, investigations of minisatellite instability in lung cancer have not been reported hitherto.

Several kinds of genomic instability, which are observed at nucleotide and chromosome levels, arise from different pathways and are involved in cancer initiation and progression. Mini- and microsatellite instabilities are representative of the former, and chromosome instability and LOH belong to the latter. Microsatellites consist of tandem repeats of short (<10 bp) units, whereas minisatellites are composed of longer repeats (10<and <100 bp) (Bois, 2003). Both are highly polymorphic showing variation between individuals. Consequently, they have found utility as an ideal marker of identification (Jeffereys *et al*, 1985). Microsatellite polymorphisms may also correlate with human pathologies (Stevanin *et al*, 2000). Furthermore, as microsatellite instability is a cause of diseases such as HNPCC, the minisatellite instability has also been associated with common cancers and hereditary diseases. Indeed, mutant alleles of HRAS1, a minisatellite locus located close to the H-ras proto-oncogene, are a major risk indicator for common types of cancer including breast, colorectal and urinary bladder carcinomas (Krontiris *et al*, 1993). In addition, there is evidence for association of type 1 diabetes with a mutated minisatellite (Bennett *et al*, 1995). Mini- and microsatellites, though consisting of similar repetitive structure, differ in mechanisms to be destabilized (Lopes *et al*, 2002). Microsatellite instability is attributed to slippage during replication, to mitotic or meiotic unequal exchanges (Sadamoto *et al*, 1994) and to DNA mismatch repair gene deficit (Fishel *et al*, 1993; Leach *et al*, 1993). On the other hand, minisatellites, targets for a mutagenic process, are destabilized through meiotic recombination (Buard *et al*, 1994), resulting in altering copy numbers of a repeat unit. Spontaneous rearrangement of minisatellites has been detected two- or three fold more frequently in the germ line than in somatic cells (Lopes *et al*, 2002). Loss of mismatch repair function is reported as not affecting minisatellite instability (Sia *et al*, 1997; Kokoska *et al*, 1999). In the present study, the incidences of mini- and microsatellite instability were low at 1.8 and 5.5%, respectively. These results imply that neither of these genetic alterations contributes to the development of most lung cancers, in contrast with their role in many cases of HNPCC (Aaltonen *et al*, 1993). The rarity of mini- and microsatellite instability also suggests that the basis of genetic alterations in lung cancer is acquired, not inherited. The frequency of microsatellite instability in lung cancer varies among different studies, ranging from 2 to 34% (Peltomaki *et al*, 1993; Ryberg *et al*, 1995; Mao *et al*, 1994; Shridhar *et al*, 1994; Fong, 1995; Miozzo *et al*, 1996). There are several possible factors for the discrepancy of the results: (i) the numbers and loci of markers examined; (ii) the length of repeat unit; (iii) the nature of the sample, that is, frozen or paraffin-embedded (Gotoh *et al*, 1999). Our experiences with analysis of frozen and paraffin-embedded tissue suggest that the alternative ways of processing the tissue before analysis are the underlying reason for the discrepancy in other studies.

As compared with the low frequency of mini- and microsatellite instability, LOH was observed in 49.1% of all cases. For ACs, the incidence was increased as the differentiation grade became less and was at its highest in p/d AC (62.5% of cases and FAL=0.188). Furthermore, it was especially high in the two anaplastic carcinomas: LCC (80%) and SCLC (100%). Hence, the results imply that the accumulation of genetic alterations contributed greatly to tumour progression, rather than initiation, of lung cancer. These findings are in line with our previous consequences by allelotyping (Tsuchiya *et al*, 1992). Among the six microsatellite markers used in this study, the loci on the region of chromosome 17p13 (TP53, HPOCA3, D17S786) demonstrated the highest frequency of LOH. So, this study confirmed again the importance of loss or inactivation at the loci in lung cancer.

Chromosome instability could induce LOH of tumour suppressor genes and/or effectively amplify oncogenes by duplicating the chromosome, resulting in tumorigenesis (Rajagopalan *et al*, 2004). The present study showed that the three subtypes, p/d AC, LCC and SCLC, had a high grade of chromosome instability as well as a high frequency of LOH (Table 4 and 6).

In conclusion, the development of lung cancer depends on genomic instability at the chromosome level rather than at the nucleotide level. Minisatellite instability appears far less important in pathogenesis of lung cancer. The significant occurrence of LOH on the same chromosomal region (17p13) suggested that the site bears a definite relationship with lung cancer development. The incidence of LOH and the grade of chromosome instability were the highest in p/d AC and anaplastic carcinomas, confirming the morphology–genotype relationship.

## Figures and Tables

**Table 1:** Clinical characteristics of patients

			**Pathological stage**				
**Subtypes**	**Gender (M:F)**	**Average age (years)**	**I**	**II**	**III**	**IV**	**Smoking index±s.d.**
w/d AC	8 : 11	62.9	9	2	5	2	359±574
m/d AC	3 : 7	60.4	3	1	6	0	414±563
p/d AC	6 : 2	64.3	3	1	3	1	619±503
SCC	9 : 1	66.9	6	0	4	0	1656±879
LCC	5 : 0	61.9	5	0	0	0	1327±563
SCLC	3 : 0	67.7	2	1	0	0	775±288

AC, adenocarcinoma; SCC, squamous cell carcinoma; LCC, large cell carcinoma; SCLC, small cell lung carcinoma; smoking index, (number of cigarettes per day) × (duration of years); w/d, well differentiated; m/d, moderately differentiated; p/d, poorly differentiated.

**Table 2:** Results of mini- and microsatellite instability, loss of heterozygosity and chromosome instability

	**Minisatellite marker**			**Microsatellite marker**						
**No.**	**D1S80**	**Apo-B**	**D17S30**	**AFM184Xe**	**D3S1067**	**D13S270**	**TP53**	**HPOCA3**	**D17S786**	**Grade of chromosome instability** ^a^
	AC, well									
1	−	−	−	−	−	−	−	−	−	0
2	−	−	−	−	−	NI	−	−	−	0
3	−	−	−	−	NI	NI	−	−	NI	0
4	−	−	−	−	−	NI	−LOH	−	−	0
5	−	−	−	−	NI	−	−	−	−	L
6	−	−	−	−	−	NI	−	−	−	H
7	−	−	−	−	−	−	−	−	−	L
8	−	−	−	−	−	NI	−	−	−	−
9	−	−	−	−	NI	−	−LOH	−	−	M
10	−	NA	−LOH	−	−	−	−	−	−	0
11	−	−	−	−	NI	−	−	−	NI	0
12	−	−	−	NI	−	NI	−	−	NI	0
13	−	−	−	−	−	−	−	−	−LOH	M
14	−	−	−	−	−	NI	−	−	−	0
15	+	+	−LOH	+	+	NI	+LOH	−LOH	+LOH	M
16	−	−	−	−	−	−	−	−	−	NP
17	−	−	−	−	−	−	−	−	−	M
18	−	−	−	−	−	NI	−	−	−	NP
19	−	−	−	−	−	−	−	−	−	NP
										
	AC, mod									
1	−	−	−	−	NI	−	−	−	−	−
2	−	−	−	−	−	−	−	−	−	−
3	−	−	−	−	−LOH	−LOH	−LOH	−LOH	−	0
4	−	−	−	−	NI	−	−	−	−	0
5	−	−	−	−	−	NI	−LOH	−LOH	NI	M
6	−	−	−	−	−	−	−	−	−	0
7	−	−	−	−	NI	−	−LOH	−	−	0
8	−	−	−	−	−LOH	−	−	−	−LOH	−
9	−	−	−	−	−	−LOH	−	−	−	0
10	−	−	−	−	+	NI	−LOH	−LOH	−LOH	M
										
	AC, por									
1	−	−	−	−LOH	−LOH	−	−	−LOH	−LOH	M
2	−	−	−	NI	−	NI	−LOH	−LOH	−	M
3	−	−	−	−	−	NI	−	−	−	M
4	−	−	−	−	−	−	−	−	−	0
5	−	−	−	−	−	−	−	−	−	NP
6	−	−	−LOH	−	NI	−	−LOH	−	NI	H
7	−	−	−LOH	−	−	−	−	−	−	M
8	−	−	−	−	−	−	−LOH	−LOH	−LOH	−
										
	SCC									
1	−	−	−	−	−	−	−	−LOH	N−I	H
2	−	−	−	−	−	−	−	−	NI	0
3	−	−	−	−	−	NI	−	−	−	0
4	−	−	−	−	NI	−	−	−	−	0
5	−	−	−	−	−	−	−LOH	−	−	M
6	−	−	−	−	NI	−	−LOH	−	−	H
7	−	−	−	−	−	NI	−	−	−	H
8	−	−	−	−	−LOH	NI	−	−	−	H
9	−	−	−	−	−	−	−	−	−	0
10	−	−	−	−	−	−	NI	−	−	NP
										
	LCC									
1	−	−	−	−	−LOH	−	−	−	−	H
2	−	−	−	−	−	−	−	−	−	0
3	−	−	−	−	−	−	−LOH	−	−	NP
4	−	−	−	−	−LOH	−	−LOH	−LOH	−LOH	M
5	−	−	−	−	−	−	−LOH	−LOH	−LOH	H
										
	SCLC									
1	−	NA	−	−	−LOH	−LOH	NI	−LOH	−LOH	M
2	−	−	−	−	−LOH	−LOH	+	−LOH	−LOH	H
3	−	−	−	−	−	NI	−LOH	−	NI	M

**Table 3:** Frequencies of mini- and microsatellite instability

	**Minisatellite instability**		**Microsatellite instability**	
**Histological subtype**	**No. of cases**	**Locus**	**No. of cases**	**locus**
w/d AC	1	Apo-B, D1S80	1	TP53, D3S1067,D17S786, AFM184Xe
m/d AC	0		1	D3S1067
p/d AC	0		0	
SCC	0		0	
LCC	0		0	
SCLC	0		1	TP53
				
No. of total (total%)	1/55(1.8%)		3/55(5.5%)	

No.: number

Minisatellite markers: Apo-B, D17S30, D1S80

Microsatellite markers: TP53, HPOCA3, D3S1067, D13S270, D17S786, AFM184Xe,

AC: adenocarcinoma; SCC: squamous cell carcinoma; LCC: large cell carcinoma; SCLC, small cell lung carcinoma; w/d, well differentiated; m/d, moderately differentiated; p/d, poorly differentiated.

**Table 4:** Histological subtypes and frequency of loss of heterozygosity and FAL

	**No. of markers showing LOH**							
**Histological subtype**	0	1	2	3	4	**No of positive cases**	**Total (%)**	**Mean FAL±s.d.**
w/d AC	14	4	0	0	1	5	26.3	0.053±0.090
m/d AC	4	2	2	1	1	6	60.0	0.180±0.199
p/d AC	3	1	2	1	1	5	62.5	0.188±0.201
SCC	6	4	0	0	0	4	40.0	0.085±0.111
LCC	1	2	0	1	1	4	80.0	0.160±0.167
SCLC	0	1	0	0	2	3	100.0	0.483±0.202

LOH, loss of heterozygosity; no., number; Total (%), number of LOH/number of informative cases; FAL, fractional allelic loss, no. of positive/total of adenocarcinoma.

**Table 5:** Number of loss of heterozygosity and the loci of mini- and microsatellite markers

	**Minisatellite markers**			**Microsatellite markers**					
	**D1S80**	**Apo-B**	**D17S30**	**AFM184Xe**	**D3S1067**	**D13S270**	**TP53**	**HPOCA3**	**D17S786**
Total no. of LOH	0	0	4	1	8	4	16	12	10
No. of NI	0	0	0	2	10	17	2	0	8
									
LOH/informative cases (%)	0.0	0.0	7.3	1.9	17.8	10.5	30.2	21.8	21.3

LOH, loss of heterozygosity; no, number; NI, not informative cases (One case of well-differentiated adenocarcinoma was not examined at the locus of Apo-B).

**Table 6:** Histological subtypes and the grade of chromosome instability

**Histological subtype**	**0**	**L**	**M**	**H**	**M+H**	**NP**
w/d AC	8	2	4	1		4
	53.3%	13.3%	26.7%	6.7%	33.4%	
m/d AC	5	0	2	0		3
	71.4%	0.0%	28.6%	0.0%	28.6%	
p/d AC	1	0	4	1		2
	16.7%	0.0%	66.7%	16.7%	83.4%	
SCC	4	0	1	4		1
	44.4%	0.0%	11.1%	44.4%	55.5%	
LCC	1	0	1	2		1
	25.0%	0.0%	25.0%	50.0%	75.0%	
SCLC	0	0	2	1		0
	0.0%	0.0%	66.7%	33.3%	100.0%	

Grading according to the number of structural alterations: L, low less than 5; M,moderate 6-15; H, high more than 16; NP, not proliferative; no., number; AC, adenocarcinoma; SCC, squamous cell carcinoma; LCC, large cell carcinoma; SCLC, small cell lung carcinoma; w/d, well differentiated; m/d, moderately differentiated; p/d, poorly differentiated.
